# A multi-point calibration method for electron probe microanalysis (EPMA) of indium in sphalerite (ZnS)

**DOI:** 10.1038/s41598-025-91085-x

**Published:** 2025-03-06

**Authors:** Thomas Schirmer, Thomas Ulrich

**Affiliations:** Institute of Disposal Research, Clausthal University of Technology, Adolph-Römer-Str. 2A, D- 38678 Clausthal-Zellerfeld, Germany

**Keywords:** Multi-point calibration, EPMA, Indium, Sphalerite, ZnS, Materials science, Chemistry, Analytical chemistry, Inorganic chemistry, Geochemistry, Geology, Mineralogy

## Abstract

This article presents a multi-point calibration approach for electron probe microanalysis (EPMA) for the trace element analysis of indium in sphalerite (ZnS). To define a multi-point calibration curve, indium and cadmium-doped ZnS crystals in a concentration range from 0 (blank) to ~ 1500 µg / g were used. The samples were measured with two different analytical settings (25 kV acceleration voltage and 100 nA beam current as well as 7 kV and 200 nA). The figures of merit including, beam stability, lower limit of detection and limit of quantification as well as the reproducibility and precision are assessed. Equally, the line overlap of Cd and chemical shift due to non-matrix matched standards is discussed. The multi-point calibration approach results in a 2–3 times improved analytical precision compared to the classical calibration approach using only one calibration sample, and detection limits down to about 20 µg / g were achieved.

## Introduction

Electron probe microanalysis (EPMA) is a relatively widely used method for non-destructive measurements of surfaces, particles and minerals with high spatial resolution^1^. Normally, the method is used to analyze major and minor elements (100 − 0.1 wt%). The quantification of trace elements (> 0.01 wt% - < 0.1 wt%) is possible with regard to detection limits, but poses a particular challenge in terms of calibration and matrix correction. In contrast to many other comparative methods, a one-point calibration is normally carried out with EPMA. For this calibration, reference materials are usually used that contain the measuring element in high concentration (as major element) and often have a completely different matrix (e.g. In in InSb) (e.g^1,2^).

The analysis of elements in low concentrations with electron probe microanalysis (EPMA) requires optimal tuning, measurement and result quantification. Electron beam and sample must be stable over an extended time because of the long measurement times (depending on the measured trace element more than 10 min) to acquire a statistically significant count number.

Additionally, the precise measurement of X-Ray count rates from elements with concentrations below 1000 µg / g with EPMA depends on a reliable measurement or calculation of the interference signal underneath the measured X-Ray line (background noise and line overlap). Also very important is the correct determination of X-ray fluorescence yield influence factors (“matrix correction”, see e.g^3^).

In order to convert the measured count rates into concentration data, an (international) reference material is required. Since the measured intensities of the characteristic X-rays generated in the sample are strongly matrix-dependent, reference materials with a comparable matrix should be used.

However, artificial alloys such as InSb, InTe or InAs are usually used to calibrate trace elements like indium in natural samples such as ZnS-containing ores (e.g^4^). In addition to the differing matrix, leading (at least) to a different spectrum curve (e.g. influencing the background count rate statistics), the element concentration of these reference materials is typically very high (48.54 wt% In for InSb) compared with the concentration in the samples (< 0.1 wt%). Due to the larger peak width at such high concentrations, it is more difficult to determine the exact peak position, which is extremely important for trace element analysis. Furthermore, the classical EPMA calibration relies on only one single reference material measurement.

The reason for this is the limited availability of international reference materials for the wide variety of compounds that can be measured by EPMA.

This article presents an approach to analyze the trace element indium in sphalerite (ZnS) using a set of artificially doped ZnS-crystals with trace concentrations of Cd and In. This method allows setting up a calibration function including precise background measurements, matrix correction and line overlap within the expected concentration range.

## Background

There are numerous publications on (trace) element analysis with EPMA. Therefore, only a selection of articles is present that cover the various aspects essential for optimizing the method development.

The following chapter lists the various factors and general measurement conditions to be considered for the analysis of trace elements using EPMA:

### Element range

In principle, all elements from Li to U can be analyzed with EPMA, whereby Li in particular has an extremely poor detection limit due to the low energy of LiKα and the low fluorescence yield. The detection limits are very strongly dependent on the matrix and it is therefore difficult to make generally valid statements. A general value of 100 µg / g for standard settings is published by^5^.

### Beam, sample stability and counting time

The measurement times for carrying out trace element analyses are very long (e.g., 360 s) compared to standard measurements (e.g. 10–20 s). Moreover, repeated measurements and a combination of spectrometers are advantageous^6,7^. The long measurement times require exact and stable beam positioning.

For higher Z elements (e.g. Ti) an increase of the kV setting leads to a decrease of the lower limit of detection (LLD) due to a better peak to background ratio. Consequently, the analysis of heavy trace element requires very high accelerating power (20–25 kV) and beam current (200–900 nA) (e.g^8–12^) to improve the counting statistics. This requires a good stability of the measurement point on sample or reference material. On the opposite, lighter elements like Al as a trace element can be measured with15 kV^3,8^.

### Line overlaps and crystal resolution

The separation of the signals of interest from unwanted spectral overlaps has to be very efficient. Therefore, the crystal resolution should be good enough to distinguish the signals within the measured angle area. A list of potential line overlaps can be determined from the wavelength tables of characteristic X-rays for the analytical crystal used (e.g^13^). A sample set can also be used to determine the line overlap. For example, a linear function with a set of reference materials (line overlap of Fe on F) is described by^14^.

### Chemical shift and determination of the exact peak position

Depending on the direct atomic / molecular environment, the energy of the characteristic X-ray radiation of the measuring element can shift. This shift can be so significant that even speciation studies are possible. For Cr this is shown by^15^. Therefore, the peak position of the measuring element on the sample can deviate from the position determined based on the theoretical angular position using the crystal verification procedure. Particularly in the case of trace element analysis, a minimal deviation of the peak position has a very large effect due to the small peak width. When checking the peak position with a highly concentrated reference material (e.g. InSb), the position cannot be determined precisely enough due to the very broad peak width. In this case, a low-concentration material (e.g. a trace element standard) is recommended.

### Background analysis and subtraction

The best way to determine the background contribution is to do an analysis of a “blank” sample that has the same or very similar matrix to the unknown sample, but does not contain the trace element to be analyzed (e .g^3^).

Multi-point measurement methods are already used for the precise determination of the background, whereby a polynomial calculation is used by measuring a large number (up to 24) of background positions instead of the linear (classical) interpolation of the background count rate at the peak position of the measuring element using one or two measurement positions^8,16^). With simulation of the spectrum underneath the element peak using a pseudo-voigt function^17^ were able to optimize their method to analyze Ta and Hf in rutile and achieved a LLD of 24 µg / g.

### Analytical volume

To estimate the penetration depth of the electrons and ratio of generated and emitted characteristic radiation Monte Carlo simulation is carried out (e.g^9^). High acceleration voltage leads to an increase in the penetration depth, and thus, to a higher probability that not only the selected grain is excited. Areas of interest for the measurement can be selected very well using the backscattered electron image with Z-contrast (BSE(Z)), for example. Although the BSE(Z) signal originates from a certain depth of the sample, any underlying grains with a different composition, but which are reached by the characteristic X-rays generated in the selected area, are not visible. Secondary fluorescence, excited by characteristic radiation from the analyzed (mineral) grain, which generates characteristic radiation in a neighboring grain with a different composition (“over-radiation”), can lead to an overdetermination of the element of interest^5,6,18^. In extreme cases, this effect can occur at a distance of up to several 100 μm (e.g^19^. , and references therein).

This means that lower kV values are better suited for a higher resolution but a higher detection limit, whereas higher kV yields a better detection limit, but with a lower spatial resolution. Lowering the excitation voltage additionally provides an opportunity to reduce the analysis volume by preventing the excitation of unneeded (or undesired) higher energy characterizing radiation.

### Sample homogeneity

The homogeneity of the samples - reference material and unknown sample - is crucial for the reproducibility of the calibration and concentration results, as the matrix correction assumes a homogeneous element distribution in the analysis volume. To determine the matrix correction factors with the required accuracy, the homogeneity of the sample must also be as high as possible, as no concentration gradients, zonings or grain boundaries can be taken into account in the calculation.

### Lower limit of detection (LLD) and limit of quantification (LOQ)

The lower limit of detection (LLD) is the measurement value of an (analytical) method up to which the analyte can still be reliably detected. This value is defined as three times the standard deviation from the measured value (e.g^20,21^) or:

1 $${\text{LLD }} = {\text{ C}}_{{{\text{Bg}}}} + {\text{ 3 }}\cdot{\text{ SDC}}_{{\text{B}}} {\text{g}}$$

where C_Bg_: Background count rate, SDC_B_g: Standard deviation of background.

Nevertheless, a reliable determination of a concentration is only possible if the LOQ is exceeded. This parameter is defined, for example, as 3.33 times the detection limit^21^). This means at a LLD of 10 µg / g a quantification is reliable > 33.3 µg / g. Optimization of the LLD is only possible with the highest possible kV and nA settings and long measurement times, whereby no greater improvement can be achieved above a certain measurement duration. This depends on the ratio of counting statistical error (sqrt of count rate) to count rate (usually logarithmic behavior, see e.g^9^). As the relative counting statistical error (i.e. in relation to the total counting rate) is only halved if the measuring time is quadrupled at a constant counting rate, extending the measuring time from this point onwards is not useful.

Typical EPMA-settings for heavier elements are up to 25 kV and starting at 200 nA up to 900 nA with measurement times of 180 s^9^.

Interestingly, the sensitivity for increasing kV settings passes through a minimum for lighter elements (e.g. Al in quartz), while a logarithmic decrease can be observed for heavier elements (e.g. Ti in quartz). For heavier elements such as Ti, the slope is so flat from about 25 kV that increasing the excitation voltage no longer brings any gain. Also, at 25 kV, increasing the current above 300 nA no improvement is achieved^8^.

Besides the instrument settings and the measurement conditions, the LLD depends on the matrix of the material. Especially for Ti, many results regarding LLD can be found. This element was therefore selected as an example to present LLD values. For example, the LLD in simple light matrices like quartz can be as low as 5 µg / g^22^). The values for Ti reported by^23^ in orthopyroxene, clinopyroxene and garnet are 6, 28 and 49 µg / g respectively. This proves that the detection sensitivity considerably depends on the matrix. Furthermore, the results published by^24^ show that the LLD of Ti in zircon could be reduced from 60 to 18 µg / g by increasing the probe current from 50 to 300 nA. Equally, detection limits of Al and Ti in quartz can be below 10 µg / g^3^). Indium LLDs at 25 kV / 60nA of 250 µg / g in stannite and stannoidite are published by^25^ and of 25 µg / g in biotite and amphibole at 20 kV / 200 nA by^4^.

### Matrix correction

In most cases, matrix correction and spectral interferences (very comprehensively discussed in^26^) have to be eliminated via a correction algorithms including factors like absorption (e.g^13^), atomic number and fluorescence (ZAF (simply: Z: atomic number, A: absorption, F: fluorescence correction factors), PAP (Pouchou and Pichoir matrix correction model^27^), X-PHI or φρ (phi-rho-zeta matrix correction model, e.g^28–30^). ). The determination of empirical correction factors is possible, but requires standards tailored to the matrix of the sample material^31^).

Special care should be taken when a trace element is to be determined in a simple matrix with a highly concentrated main element. In that case, subordinate lines can also trigger secondary fluorescence (e.g. Excitation of MnKα by FeKβ in steel^32^).

### Calibration strategies

Normally, a single (international) reference material with the elements of interest is used to calibrate the electron microprobe analysis. The reference samples are cross checked with other methods like LA-ICP-MS, SIMS or bulk methods like XRF, ICP-MS or ICP-OES^9^). The reference materials often do not correspond to the matrix of the unknown sample to be measured (e.g., InSb for In in ZnS). Multi-point calibrations are not common because the matrices of the grains are normally very different, and it is difficult to find reference materials with the same matrix and different contents of the element of interest (especially with traces).

Multi-point calibrations for minor compounds in steel (C, Si, Mn) are presented by^33^. It was shown that increasing the current from 100 to 300 nA significantly improves the slope of the calibration line and triples the sensitivity of the method. An important aspect here - especially with lighter elements - is the contamination with carbon by the measurement (e.g., when recording electron images). The accuracy of the results is checked by comparison with other methods (e.g., XRF) (e.g^11^), where the results for all measured elements in the comparative diagram ideally align along a 45° line.

## Materials and methods

### Instrument setup and analytical conditions

For the analysis of In in sphalerite we used a Cameca SXFIVE FE (Field Emission) electron probe (CAMECA SAS, Gennevilliers Cedex, France). The acceleration voltage was 25 kV at 100 nA and 7 kV at 200 nA, respectively. The beam size parameter in the instrument software was set to 0, which leads to the smallest possible adjustable beam diameter. This diameter should substantial below 1 μm (100–600 nm with field emitters of Schottky-type^34^)). Indium (Lα) was measured on three wavelength dispersive spectrometers (WDX) equipped with two large PET and one standard PET crystal (PET: pentaerythritol). Additionally the elements S, Zn, and Cd were determined. The count rates (peak and background) for indium were averaged over all spectrometers. In the classical (single point) calibration the X-PHI-Model^35^) was applied for the calculation of In concentrations. For the classical calibration of S (Kα), Zn (Kα), Cd (Lα) and In (Lα) pure ZnS, pure CdS and pure InSb were used. The reference materials were provided by P&H Developments, The Shire 85 A Simmondley village, Glossop, Derbyshire, UK. The measurement conditions are presented in Table 1.

**Table 1:** Measurement conditions (part of a general measurement program) and lower limit of detections. OVL: overlapping line, LLD: detection limit (determined by instrument software), given in Μg/g.

Line 25 / 7 kV	Reference	Xtal	Peak (s)	Bkg1 (s)	Bkg2 (s)	LLD 25 kV	LLD 7 kV
InLα / InLα	InSb	LPET	300	OVL CdLβ	170	25	110
InLα / In Lα	InSb	PET	410	OVL CdLβ	70		
InLα / InLα	InSb	LPET	470	OVL CdLβ	70		
SKα / SKα	ZnS	LPET	20	10	10	240	241
ZnKα / ZnLα	ZnS	LiF	60	10	10	275	416
CdLα / CdLα	CdS	LPET	30	15	15	119	315

### Preliminary considerations

Based on the information found in the literature summarized above the following first considerations can be made. The energy of InLα (3.286 keV) is high enough, that an increase of acceleration voltage and beam current should lead to higher sensitivity, and therefore, to a lower LLD. Excitation settings at 25 kV / 100 nA should deliver sufficient sensitivity, but the stability of the samples must be investigated. A combination three spectrometers arranged at different positions around the sample is used. A line overlap survey identified Cd as the most important interference (CdKα). By using the same matrix of the reference material (i.e. In-doped ZnS) as the sphalerite to be measured, a chemical shift of InLα between reference material and sample can be neglected. The background count rate can be reliably determined on pure ZnS reference material so that matrix correction can also be omitted.

All used samples (references and unknowns) are artificially grown crystals, consequently, very good homogeneity should be expected. The LOQ (LLD * 3.3; for LLD see Table 1) for In is 83 µg / g (25 kV) and 367 µg / g (7 kV) should be low enough for a reliable measurement in the targeted concentration range. Zn Kα / Kβ are the only major element lines that can excite In-Lα. However, for both lines the absorption coefficients are low (no absorption edges nearby, see e.g^13^). At 7 kV matrix correction for Zn Kα / Kβ (8.63 / 9.57 keV) is not necessary because this setting, leading to a maximum of 7 keV is not sufficient to excite these lines.

For the method development, penetration depth of the electrons and analysis depth of the characteristic X-ray lines, line overlaps, background count rate and matrix effects, must be taken into consideration. This is important to estimate the spatial resolution of the method. To carry out matrix and line overlap correction the Lα line of Cd as the main line overlapping element and the typical matrix elements Zn (Kα) and S (Kα) were taken into account. Using a general formula, presented in^36^), the penetration depth (P_d_) of the electron beam in ZnS with an average density of 4.05 g / cm^3^ was calculated to be 3.09 μm at 25 kV and 0.46 μm at 7 kV:

2 $$\tt \normalsize \tt \normalsize {\text{P}}_{{\text{d}}} \left( {\mu {\text{m}}} \right){\text{ }} = {\text{ }}\left( {0.{\text{1 }}\cdot{\text{U(kV)}}^{{{\text{1}}.{\text{5}}}} } \right){\text{ }}/{\text{ d }}\left( {{\text{g }}/{\text{cm}}^{{\text{3}}} } \right),{\text{ d }} = {\text{ density of absorber }}\left( {{\text{g }}/{\text{cm}}^{{\text{3}}} } \right){\text{, U }} = {\text{ accelerating voltage}}\left( {{\text{kV}}} \right)$$

Using the average density and the mass absorption coefficients (MAC, µ / ρ, cm^2^ / g)^13^) of In (Lα) in Zn and S, the escape depth (D_e_), defined as the emission of 1% initial intensity I at the surface can be estimated using the Lambert Beers law:

3 $${\text{I}} = {\text{I}}_{0} \left( {{\text{e}}^{{ - {\text{MAC}}\cdot{\text{d}}\cdot{\text{t}}}} } \right),{\text{ d }} = {\text{ density of absorber }}\left( {{\text{g }}/{\text{cm}}^{{\text{3}}} } \right),{\text{ t }} = {\text{ depth }}\left( {{\text{cm}}} \right)$$

In ZnS D_e_ for In (Lα, 3.286 keV) is ~ 13 μm, for Cd (Lα, 3.133 keV) ~ 10 μm, for S (Kα,2.307 keV) ~ 8 μm and for the high energy Zn radiation (Kα, 8.368 keV) ~ 140 μm. Therefore, the penetration depth of the electron beam is not the limiting depth for the excitation of In and Cd. At 25 kV the electron beam excites Zn(Kα), which will secondary excite In(Lα) and Cd(Lα) on its path into the sample. The maximum penetration depth is P_d_ + D_e_ = ~ 143 μm. As a result, some of In(Lα) radiation will not originate from a depth of 3.09 μm but from ~ 13 μm (the D_e_ for In Lα). The same applies to part of the Cd(Lα) radiation which will originate from ~ 10 μm. At 7 kV no Zn(Kα) will be produced so that both In(Lα) and Cd(Lα) are coming from a maximum depth of about 0.46 μm which lead to a distinctively better spatial resolution. Voltages below 7 kV are not advantageous because of too low ionization cross sections^34^) and inferior excitation efficiency for InLα. As a rule of thumb, voltage settings (corresponding to the maximum acceleration energy (MaxkeV) of the electrons: kV_Setting_ ~ MaxkeV_electrons_) below 2-times the keV of the measurement line (for InLα: 2·3.286 = 6.57) deliver not sufficient excitation energy.

**Fig. 1:**
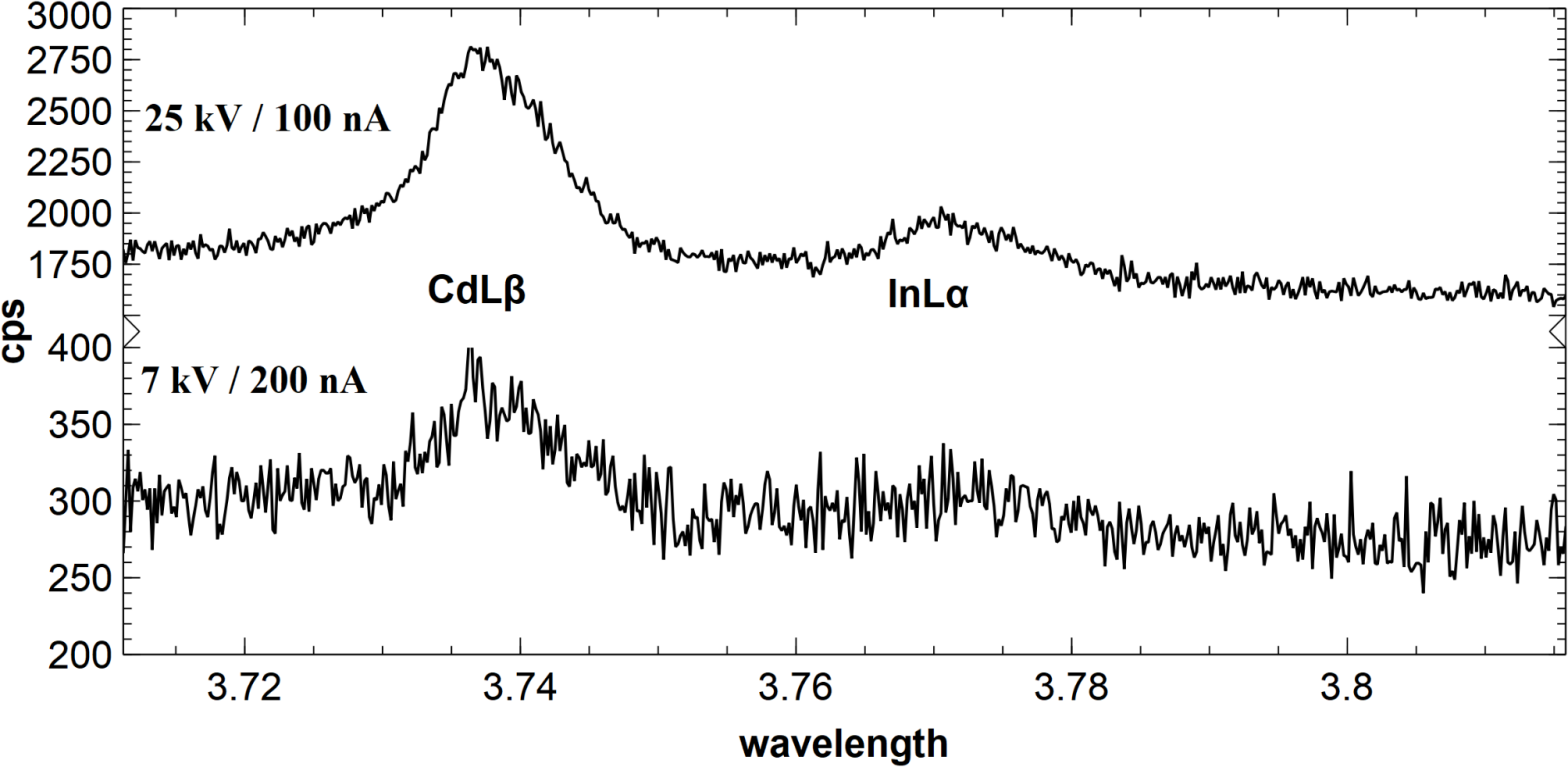
Fast WDX scan, calculated as the sum of three spectrometers (2xLPET + 1xPET) of the wavelength range of the InLa peak with 25 kV / 100 nA (above) and 7 kV / 200 nA (below) of a ZnS: In with 447 µg / g In and 3440 µg / g Cd. The count rate at 25 kV is distinctively higher.

Based on these considerations, two excitation settings were selected: 25 kV at 100 nA and 7 kV at 200 nA. The first setting stands for high intensity (best detection limit), the second for high resolution and minimal matrix effects (omitting ZnKα generation). Nevertheless, the high electron density at a small sampling volume will impact the samples. Due to the more than 2000 times larger analytical volume and the considerably higher energy of the electrons, the count rate for InLα at 25 kV is distinctively higher compared with 7 kV (Fig. 1, also discussed in^34^).

The peak count rates per second (cps) on a ZnS sample with 447 µg / g indium differ by a factor of 8.5 between 25 kV / 200 nA and 7 kV / 200 nA. The peak / background ratio at 25 kV / 200 nA and at 7 kV / 200 nA is very similar (5.8 and 5.9), but the relative standard deviation of the count rates on peak and background is distinctively higher at 7 kV (Table 2).

**Table 2:** Statistics on peak count rates for 25 kV / 200 nA and 7 kV / 200 nA. Sp_Ave: averaged spectrometer counts, peak Ave: average peak / averaged spectrometers, peak rel. Dev. rel.tive standard deviation for peak.

LPET (1)	PET (2)	LPET (3)	Sp_Ave	LPET (1)	PET (2)	LPET (3)	Sp_Ave
25 kV / 200 nA, cps				7 kV / 200 nA, cps			
Peak, 300s				Peak, 300s			
138	38	140	126	16	4	15	14
140	37	142	129	17	4	17	15
138	36	143	128	17	4	16	15
135	35	146	126	19	5	18	18
138	38	139	125	13	4	19	13
**Peak Ave**			**127**	**Peak Ave**			**15**
**Peak Rel StDev.**			**1.2**	**Peak Rel StDev.**			**12.4**
**Ratio Peak cps 25 kV / 7 kV: 8.47**							

### Materials

For the calibration of the instrument 10 platelet-shaped, synthetically grown ZnS samples with a diameter of 5 mm and 0.1 mm thickness were used. They were produced at Miracrys LLC (Miracrys LLC, Nizhny Novgorod, ul Gazovskaya, 11 Suite 72).

The goal was to obtain a suite of ZnS reference materials with different In concentrations, ranging from 0 to 1500 µg / g, while Cd as main overlapping element should have a constant concentration. Half of each reference sample was analyzed with inductively coupled plasma optical emission spectroscopy (ICP-OES) to determine the precise concentrations of Cd and In in the reference samples (Table 3). The measurements were carried out with an Agilent ICP-OES 5100 VDV system on acid digestions with HCl using a hot plate. The used wavelengths were 214,439 nm for Cd and 230,606 nm for In. It turned out, that the concentrations of Cd and In in the 10 samples were not as evenly distributed as hoped for. Nevertheless, the range in concentration is sufficient to obtain a calibration curve.

The remaining pieces of the reference materials were embedded upright with araldite epoxy resin for the electron microprobe analysis. Seven samples were used for the calibration curve and three samples were treated as unknowns. For quantification of the results and to test precision and accuracy, InSb as the reference material for the classical calibration and the new multi-point calibration was used.

**Table 3:** Cd and in concentrations measured with ICP-OES of the synthetically crystallized and doped ZnS reference material.

Element	Cd	In
Unit	RefConc. (µg / g)	
Cal1	8864	< 0.01
Cal2	3440	447
Cal3	269	588
Cal4	319	601
Cal5	295	699
Cal6	13	1025
Cal7	1791	1460
*Ref2*	258	577
*Ref1*	323	654
*Ref3*	818	1344

Seven samples (Cal1-Cal7) were used to form a calibration curve to calibrate the SXFIVE FE. The samples Ref1 – Ref3 were treated as unknowns to test the accuracy and precision of the multi-point calibration approach. LLD for in measured with ICP-OES: <0.01 µg / G, as taken from^37^). The relative standard deviation in percent is: Cd: 0.45% and In: 2.2%.

## Results

### Sample stability

**Fig. 2:**
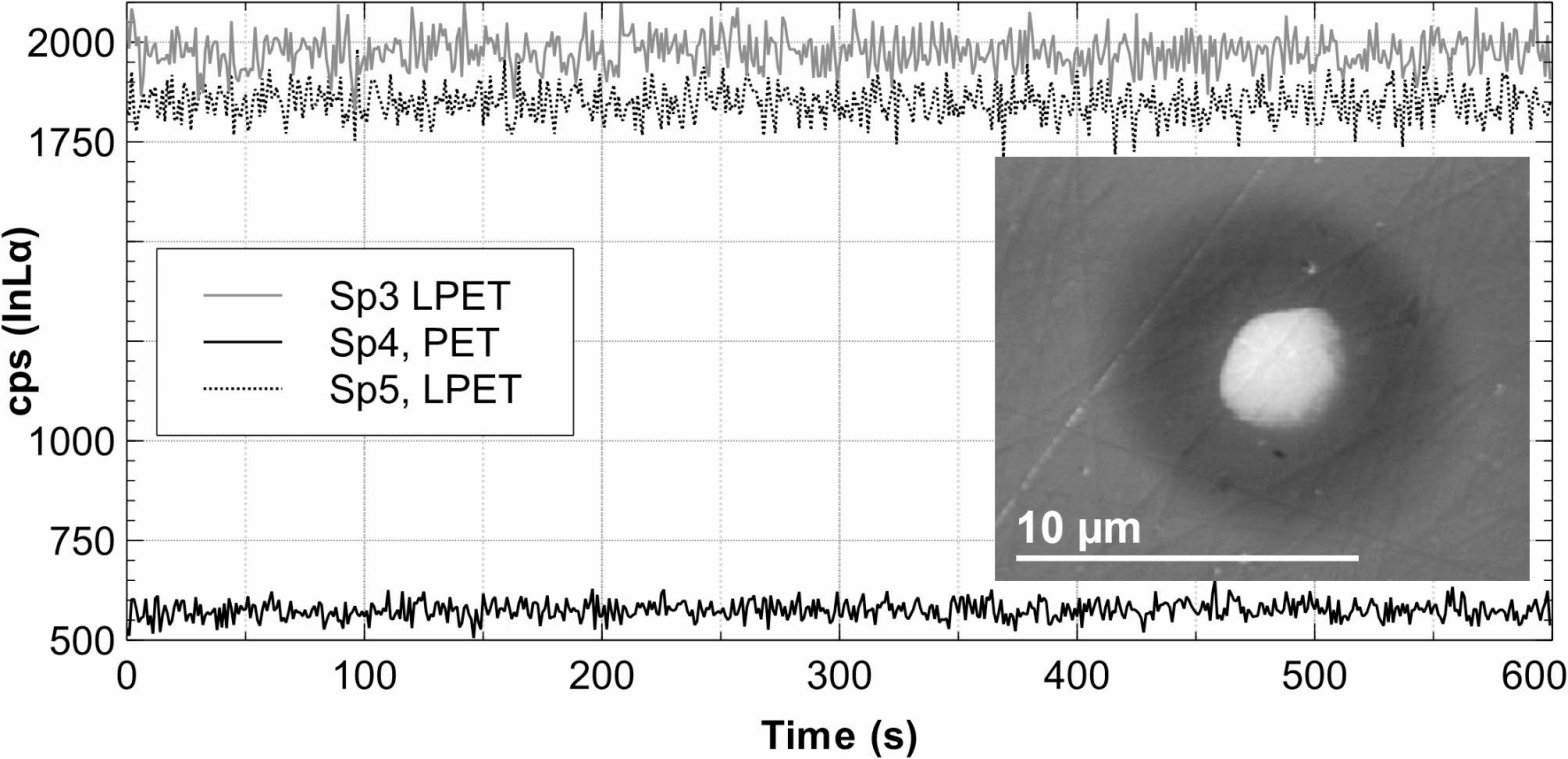
Count rates for InLα on three spectrometers (Sp3, Sp4, SP5) on crystals LPET (Large-PET) and standard PET (PET) for 600 s at 25 kV / 100 nA on a ZnS test sample nominally doped with In (1000 µg / g) and Cd (5000 µg / g). The signals show no measurable time shift. The secondary (SE) electron micrograph (5 μm) shows some contamination probably implanted by the beam but virtually no deterioration as the scratches on the sample through the measurement point remain visible.

**Fig. 3:**
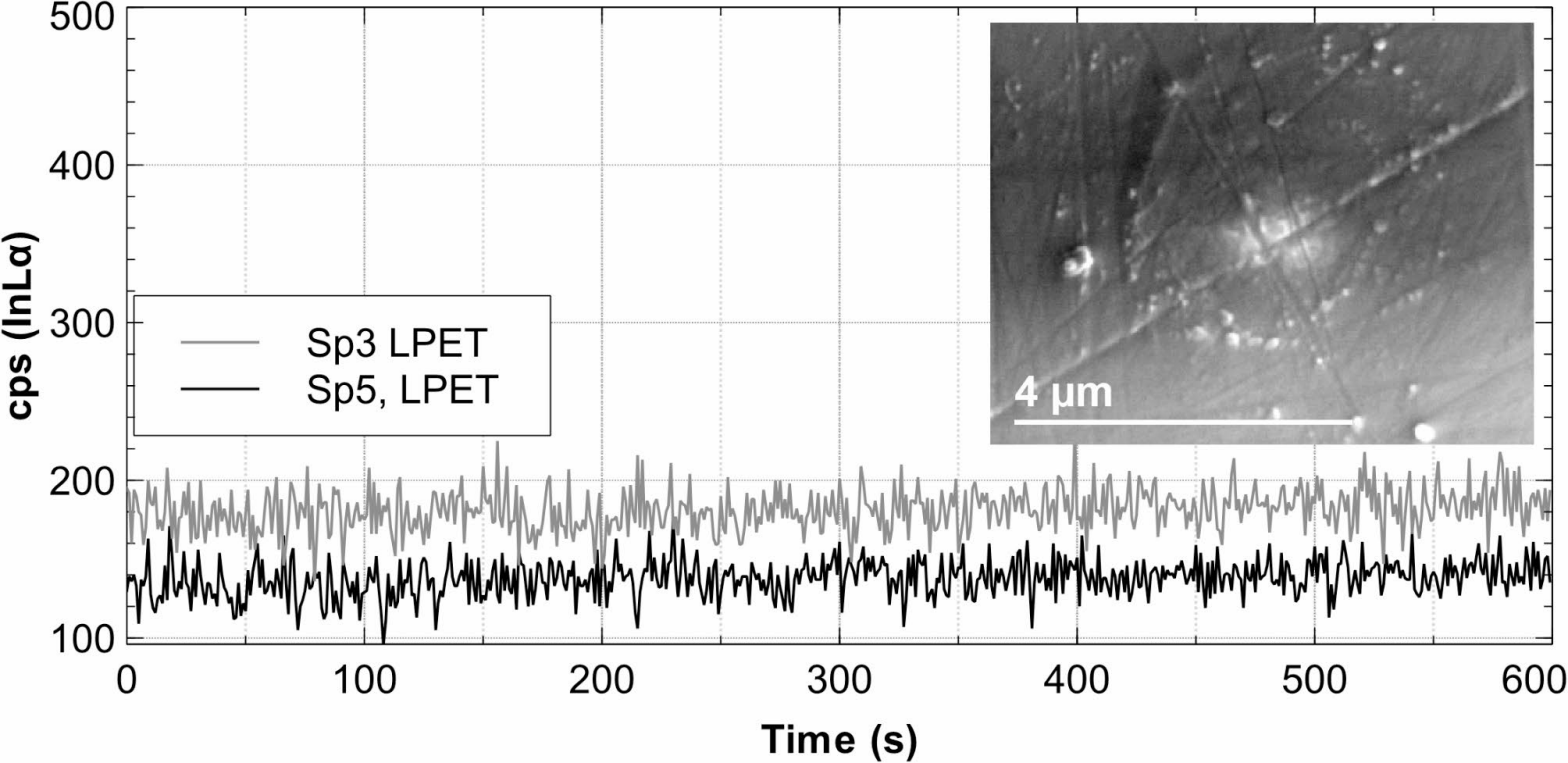
Count rates for InLα on two spectrometers (Sp3, Sp5) on crystals LPET (Large-PET) for 600 s at 7 kV / 200 nA on a ZnS test sample nominally doped with In (1000 µg / g) and Cd (5000 µg / g). The signals show no measurable time shift. The secondary (SE) electron micrograph (5 μm) shows some contamination but virtually no deterioration, as the scratches on the sample through the measurement point remain visible.

For trace element analysis, the count rate should not drift during the measurement. Potential drift was tested with a doped ZnS test sample that contained nominally 1000 µg / g In and 5000 µg / g Cd. It was measured for 600 s at 7 kV / 200 nA and 25 kV / 100 nA (Figs. 2 and 3).

The secondary electron (SE) micrograph of the spots of analysis shows a slight buildup of contamination, probably implanted by the electron beam but no deterioration (Figs. 2 and 3).

### Calibration

#### Calibration with InSb

The classical procedure was carried out via measurement of the count rates for background and peak on a referenced InSb grain. These counts per second values are transferred into a quantitative result using the count rates of the element of interest, its concentration in the reference material, and the matrix correction.

#### Multi-point calibration

**Fig. 4:**
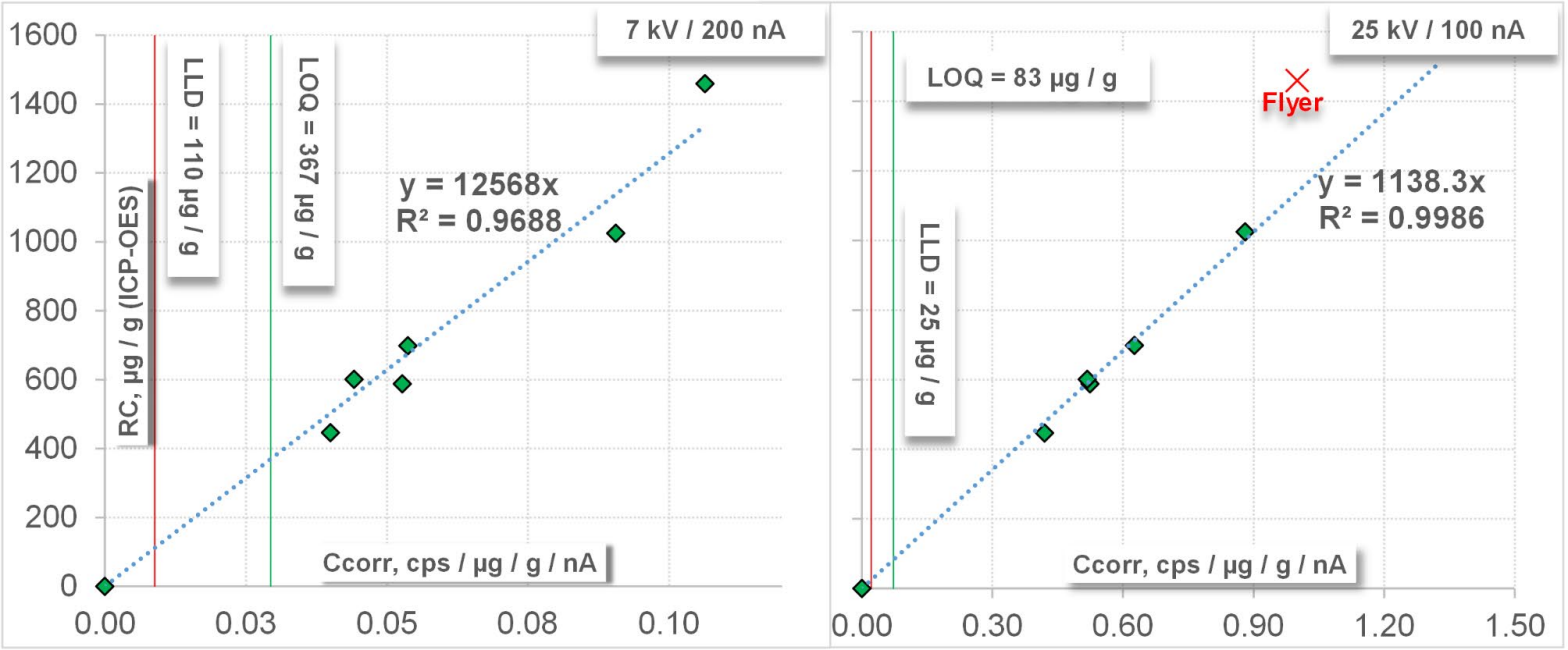
Calibration plots for InLα, measured with 7 kV / 200 nA (left) and 25 kV / 100 nA (right), Repeats *n* = 5. LLD: lower limit of detection as average over all measurements (5*7 = 35 each) determined by instrument software, LOQ: Limit of quantification (based on Eq. 1, Chap. 2.8), Flyer: Standard removed from calibration, RC: Reference concentration, determined with ICP-OES. Standard error 7 kV / 200 nA (left): 86.9, standard error 25 kV / 100 nA (right): 18.4.

For the calculation of the multi-point calibration count rates cps / µg / g / nA preprocessed by the Cameca instrument software were used. The background on a pure sphalerite sample was set as zero reference. The In concentrations of the reference samples (see Table 3, Ref1 -3) samples were determined as follows:

4 $$\left[ {{\text{In}}} \right]_{{\mu {\text{g}}/{\text{g}}}} = {\text{ Z}} - \left[ {{\text{Z}}_{{{\text{Bkg}}}} + \left( {{\text{F}}_{{{\text{Cd}}}} \cdot{\text{C}}_{{{\text{Cd}}}} } \right)} \right]\cdot{\text{F}}_{{{\text{sl}}}}$$

(Z: Count rate (cps / µg / g / nA), Z_Bkg_: Background count rate (cps / µg / g / nA), F_Cd_: Cd line overlap factor, C_Cd_: Cd concentration in the reference material, F_sl_: Slope of calibration as determined in Fig. 4. The contribution of the CdLα spectral line overlap was calculated using the In-free ZnS: Cd initial sample Cal1 (Table 3):

5 $${\text{F}}_{{{\text{Cd}}}} = {\text{ }}\left( {{\text{Z}} - {\text{Z}}_{{{\text{Bkg}}}} } \right)\cdot{\text{C}}_{{{\text{Cd}}}}$$

(F_Cd_: Cd line overlap factor, Z: Count rate (cps / µg / g / nA), Z_Bkg_: Background count rate (cps / µg / g / nA), C_Cd_: Cd concentration in reference material.

The linear equation was forced through zero, because the blank concentration was already subtracted.

With this calibration, it was possible to reduce the deviation of the measured indium concentrations from the reference values to below ± 10% (Table 4). This is particular the case for lower concentrations (< 1000 µg / g) and the lower kV setting.

### Precision and reproducibility for the reference samples

To verify and compare the approach of using a multi-point calibration, the results of the EPMA analyses of the three samples (Ref1-Ref3) where compared to the concentration values that were determined by ICP-OES for these samples. The detection limit for indium with ICP-OES as given by^37^ is below 0.01 µg / g and therefore more than 10^3^- to 10^4^-times better than for EPMA with the calculated average detection limit of about 18 µg / g at 25 kV / 100 nA and 110 µg / g at 7 kV / 200 nA, respectively. Nevertheless, for a reliable statement of concentration values, the limit of quantification must be exceeded. In this case, this is ~ 83 µg / g (3.3*25) at 25 kV / 100 nA and ~ 367 µg / g (3.3*110) at 7 kV / 200 nA. In order to document the residual deviation (scattering) of the multi-point calibration and to compare these values with the classical calibration (where these samples are not used for calibration), the calibration standards were back-calculated using the determined calibration function. The results are presented in Table 4. In the set of measured values for the 25 kV / 100 nA calibration, a sample (“flyer”) had to be removed from the calculation of the calibration function. This sample (“Cal7”, Table 4) also provided fluctuating and deviating values in the classic calibration. This is possibly due to particularly high inhomogeneities on the measurement surface of this specific sample.

An In-free sample doped with 8860 µg / g cadmium (determined with ICP-OES) resulted in indium equivalent concentrations of 54 µg / g at 25 kV / 100 nA and 15 µg / g at 7 kV / 200 nA, resulting from the overlapping element Cd. Using the classical calibration the values were 136 and 142 µg / g, respectively. Equally, the deviation of the measured concentrations compared to the reference values is much lower when using the multi-point calibration compared to the classical calibration (Table 4). It can be seen that the classical calibration method yields up to 30% deviation with a medium systematic error of about 20%, while the multi-point calibration reproduces the references values to ± 10% relative (Table 4). The arithmetic averages of the reference samples Ref1–3 of the reproducibility “R” are quite close to 100% (101.9 and 101.3, resp.) with a systematic error of < 2% (see “Systematical Average” in Table 4).

**Table 4:**
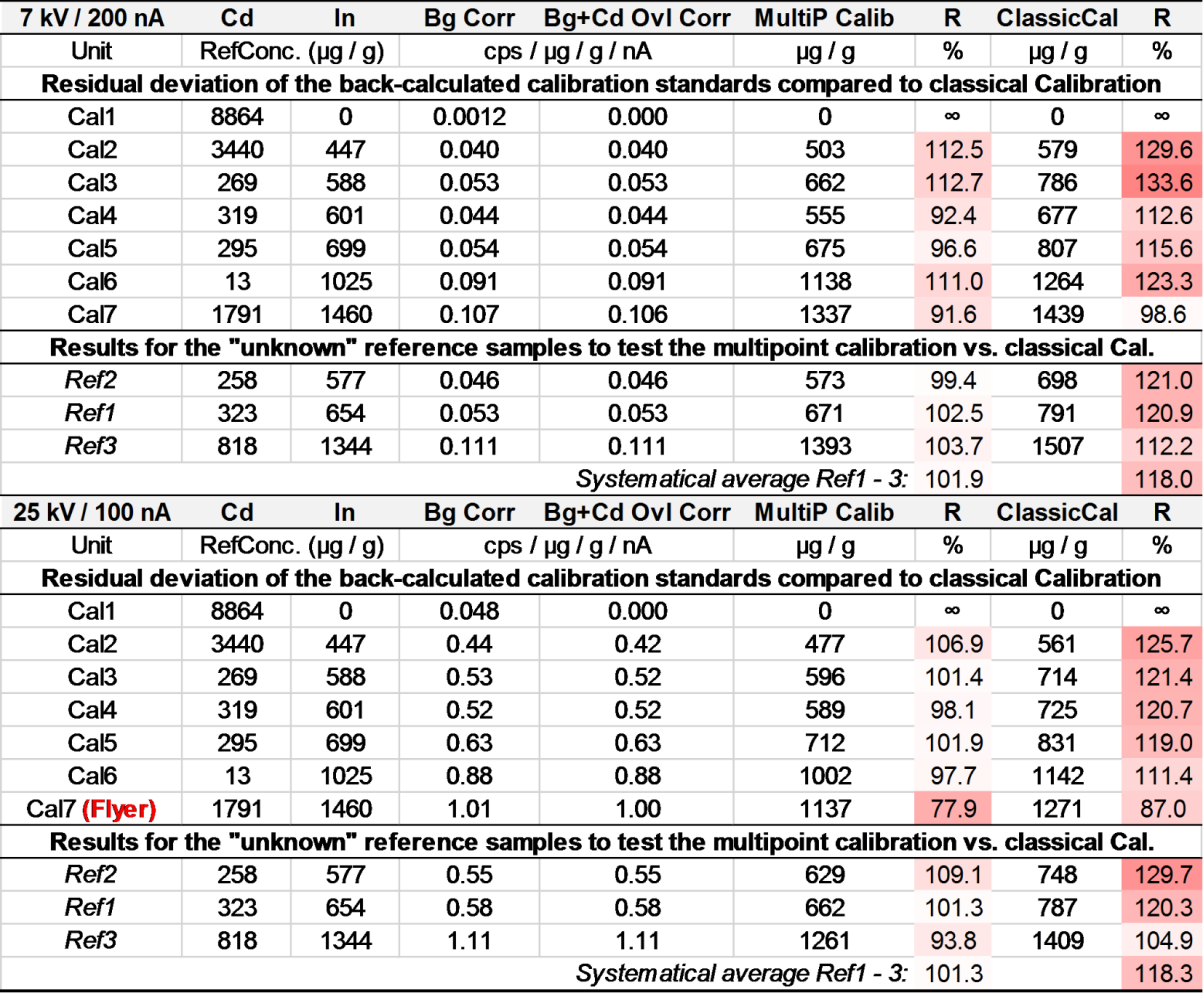
Comparison of multi-point calibration and classical calibration for 7 kV / 200 nA and 25 kV / 100 nA (10 samples, repeats: *n* = 5). RefConc: determined with ICP-OES, Bg Corr: background corrected values, Bg + cd Ovl Corr: background and cd line overlap corrected values, multip calib and classical Cal: concentrations determined with multi-point calibration and classical calibration, respectively, R: Measured / reference concentration. Flyer: excluded from calibration curve (see Fig. 4). The relative standard deviation in percent for In is 7.3% for 7 kV / 200 nA and 2.2% for 25 kV / 100 nA.

## Discussion

The motivation for this study was the determination of In in sphalerite from a syn-sedimentary exhalative deposit, whose In contents was expected to be not much higher than 600 µg / g due to the lack of Cu for a diadochic replacement according to 2 Zn^2+^ --> Cu^+^ + In^3+^^38^). Although it was possible to analyze In in the expected concentration range with the classical calibration in terms of LOD, it was necessary to check if there was any systematic variation in the values measured. In addition, the minerals in the analyzed samples were intensely intergrown, so a chemical resolution of a few µm was required. Moreover, there was a possibility that the classical calibration based on comparing the count rates of an element (indium) in a reference material with a high concentration of In (e.g. InSb with 48.54 wt% In) would not provide the best accuracy. Because of the strong matrix dependency of the used quantification method (XRF with electron excitation), also the difference in the chemical composition has to be taken into account. There existed a distinct difference between reference material and sample – in this case InSb and sphalerite. This led to the idea of producing a set of indium and cadmium-bearing sphalerite reference samples with a concentration range and a similar matrix (e.g. by addition of Cd as the main overlapping element).

Synthetically produced reference materials must fulfill the following criteria:

1. Show a broad concentration range to derive a calibration function.
2. Be homogeneous at the micrometer scale.
3. Be stable under a high current of the electron beam.

The composition of the reference material must be reliably determined using a different (and much more sensitive) method. In this case, ICP-OES was selected. These results revealed that the measured concentrations among the different samples were not as evenly distributed as desired over the planned concentration range of 200–1800 µg indium (see Fig. 4). Therefore, the second criterion was not optimally fulfilled. The homogeneity was tested with repeated measurements. The 50 measurements (5 repeats and 10 samples, see Table 3) with the setting of 25 kV / 100 nA, yield better sensitivity than the analyses with 7 kV / 200 nA, and the mean relative standard deviation is below 3%. Therefore, the homogeneity can be assessed as very good (except for sample Cal7, see Table 4 and explanations in the text). The stability under the electron beam as shown in Figs. 2 and 3 is very good. During the measurement, the count rates were very stable (no shift with time) and the SE images show no cratering (Figs. 2 and 3). As expected, the correlation coefficient of the linear regression is significantly better at 25 kV / 100 nA than at 7 kV / 200 nA. The slope in the latter case is about 10 times higher, which indicates the considerably lower sensitivity. Using a multi-point calibration involves considerably more work compared to the classical calibration approach and is only worthwhile applying if a specific trace element must be analyzed with exceptional accuracy.

## Conclusions and outlook

Our investigations show that analytical precision can be improved with a multi-point calibration. The concentrations of the reference materials were still well above the calculated detection limit. Future reference materials for calibration should contain also concentrations between the detection limit and the limit of quantification (LOQ) (110–367 µg / g for 7 kV / 200 nA and 25–83 µg / g for 25 kV / 100 nA), which could help to lower the detection limit even further by better describing the lowest possible measurable concentration range. The selected target concentrations and the desired concentration range could not quite be achieved with the crystal growing method used. This poses a challenge for the production of crystals, which still needs to be overcome. Stable indium-doped glasses with high Zn and S contents could also be used. However, to obtain the homogeneity necessary for microanalysis of trace elements can be a problem, depending on the viscosity of the glass melt. A sol-gel process could provide an alternative solution. Initial tests with lithium tetraborate fused pellets (which would be a simple and widely available method) have shown that the stability of the material is, however, not suitable for electron beam analysis.

## Data Availability

The datasets used and/or analysed during the current study available from the corresponding author on reasonable request. The data should not be circulated without the knowledge of the authors, as they were created as part of a funding from the German Federal Ministry of Education and Research.
